# Machine learning: predicting lymph node metastasis around the entrance point to the recurrent laryngeal nerve in cN0 papillary thyroid carcinoma

**DOI:** 10.3389/fendo.2026.1721148

**Published:** 2026-03-02

**Authors:** Jie Peng, Jing Zhou, Yuping Deng, Qian Xiao, Xinliang Su, Chang Deng

**Affiliations:** 1 Department of Breast and Thyroid Surgery, The First Affiliated Hospital of Chongqing Medical University, Chongqing, China; 2 Department of Breast and Thyroid, Women and Children’s Hospital of Chongqing Medical University: Chongqing Health Center for Women and Children, Chongqing, China; 3 Department of Breast and Thyroid Surgery, The Seventh People’s Hospital of Chongqing, Chongqing, China

**Keywords:** clinical lymph node negative, lymph nodes around the entrance point of the recurrent laryngeal nerve, machine learning, papillary thyroid carcinoma, Shapley additive explanations

## Abstract

**Background:**

Owing to the limited characterization of lymph nodes around the entrance point of the recurrent laryngeal nerve (LN-epRLN) in clinical lymph node negative (cN0) papillary thyroid carcinoma (PTC), this study sought to develop machine learning (ML) models to predict LN-epRLN metastasis, identify the optimal model, and improve interpretability using explainable artificial intelligence techniques.

**Methods:**

We retrospectively reviewed 1,800 patients with cN0-PTC who underwent central lymph node dissection (CLND) with systematic LN-epRLN sampling. Histopathological evaluation confirmed metastatic status. Patients were randomly divided into training and testing sets at a 7:3 ratio. Nine ML models were constructed and optimized through 10-fold cross-validation and grid search. Performance was assessed using 11 metrics, including AUC, accuracy, sensitivity, and specificity. The best-performing model was compared against traditional nomograms via probability-based ranking analysis (PMRA).

**Results:**

LN-epRLNs were identified in 149 out of 1800 PTC patients, with a metastasis rate of 19.46%. The Random Forest (RF) model outperformed others, achieving training/testing scores of 0.914/0.911 accuracy, 0.956/0.919 AUC, 0.993/0.974 specificity, and 0.609/0.500 sensitivity. A simplified model incorporating seven key predictors—total central lymph node metastasis number and ratio, pretracheal lymph node metastasis number and ratio, tumor size, age, and paratracheal lymph node metastasis number—retained high predictive performance. SHAPley Additive exPlanations (SHAP) analysis highlighted central compartment metastasis burden (number and ratio) as the most influential predictors.

**Conclusion:**

The interpretable ML model developed in this study, leveraging the RF, provides a reliable tool for preoperative and intraoperative prediction of LN-epRLN metastasis in cN0 PTC patients. This approach has the potential to guide individualized surgical planning, optimizing the balance between oncological resection completeness and functional preservation.

## 1 Introduction

Thyroid cancer incidence continues to rise globally (1, 2). In China, it ranked as the third most common malignancy in 2022, accounting for 9.7% of all new cancer cases, and was the third most frequently diagnosed cancer among women (14.9%) (2–4). Papillary thyroid carcinoma (PTC) represents approximately 90% of all thyroid cancers (5). Although PTC generally carries a favorable prognosis, it exhibits a pronounced tendency for lymph node metastasis (LNM), with rates ranging from 20% to 90%, and a recurrence rate approaching 30% (6, 7). LNM is a well-established risk factor for recurrence and is associated not only with reduced survival but also with complexities in surgical management and follow-up (1, 8).

Current surgical management of clinical lymph node negative (cN0) PTC is evolving towards a more precise, subregion-based approach to central lymph node dissection (CLND) (1, 9, 10). This shift is driven by the recognition of varying metastatic risks in different anatomical subcompartments (11–13). For instance, metastasis to lymph nodes posterior to the right recurrent laryngeal nerve (LN-prRLN) has been reported in 9.36% to 38.27% of PTC cases (14–16). Similarly, the lymph nodes situated around the point where the recurrent laryngeal nerve enters the larynx (LN-epRLN) represent another critical subregion within the central compartment (17, 18). In early studies, it was referred to as the paraesophageal lymph node (19, 20). However, the clinical significance and metastatic patterns of LN-epRLN remain poorly characterized in the literature (17, 21).

Dissection of the central compartment, particularly around the recurrent laryngeal nerve (RLN), carries significant risks, including permanent injury to the RLN and hypoparathyroidism (22, 23). The LN-epRLN region poses a unique surgical challenge due to its intimate anatomical relationship with the nerve, increasing the risk of iatrogenic injury during dissection. Current Chinese guidelines recommend unilateral lobectomy with ipsilateral prophylactic CLND as a standard surgical approach for cN0-PTC (4). Nevertheless, the decision to perform a comprehensive dissection versus a more selective approach in the high-risk LN-epRLN area remains a surgical dilemma, balancing oncological completeness against functional preservation (17, 21).

Conventional statistical methods often fall short in integrating multimodal clinical data and capturing complex, non-linear interactions among predictive variables. In this context, artificial intelligence (AI), particularly ML, offers a powerful alternative for developing robust predictive models by learning from high-dimensional datasets and identifying subtle patterns beyond the reach of traditional approaches (24, 25). To address this challenge, we leveraged machine learning (ML) to develop and validate predictive models for LN-epRLN metastasis (LNM-epRLN). The primary objectives of this study were to identify the optimal ML model for predicting LNM-epRLN, to simplify the model by identifying the most critical predictive features, and to enhance the interpretability of the model using SHapley Additive exPlanations (SHAP) to elucidate feature contributions (26). We aimed to provide a data-driven tool to facilitate individualized and precise intraoperative decision-making regarding LN-epRLN dissection.

## 2 Materials and methods

### 2.1 Patient inclusion and data processing

**Figure 1:**
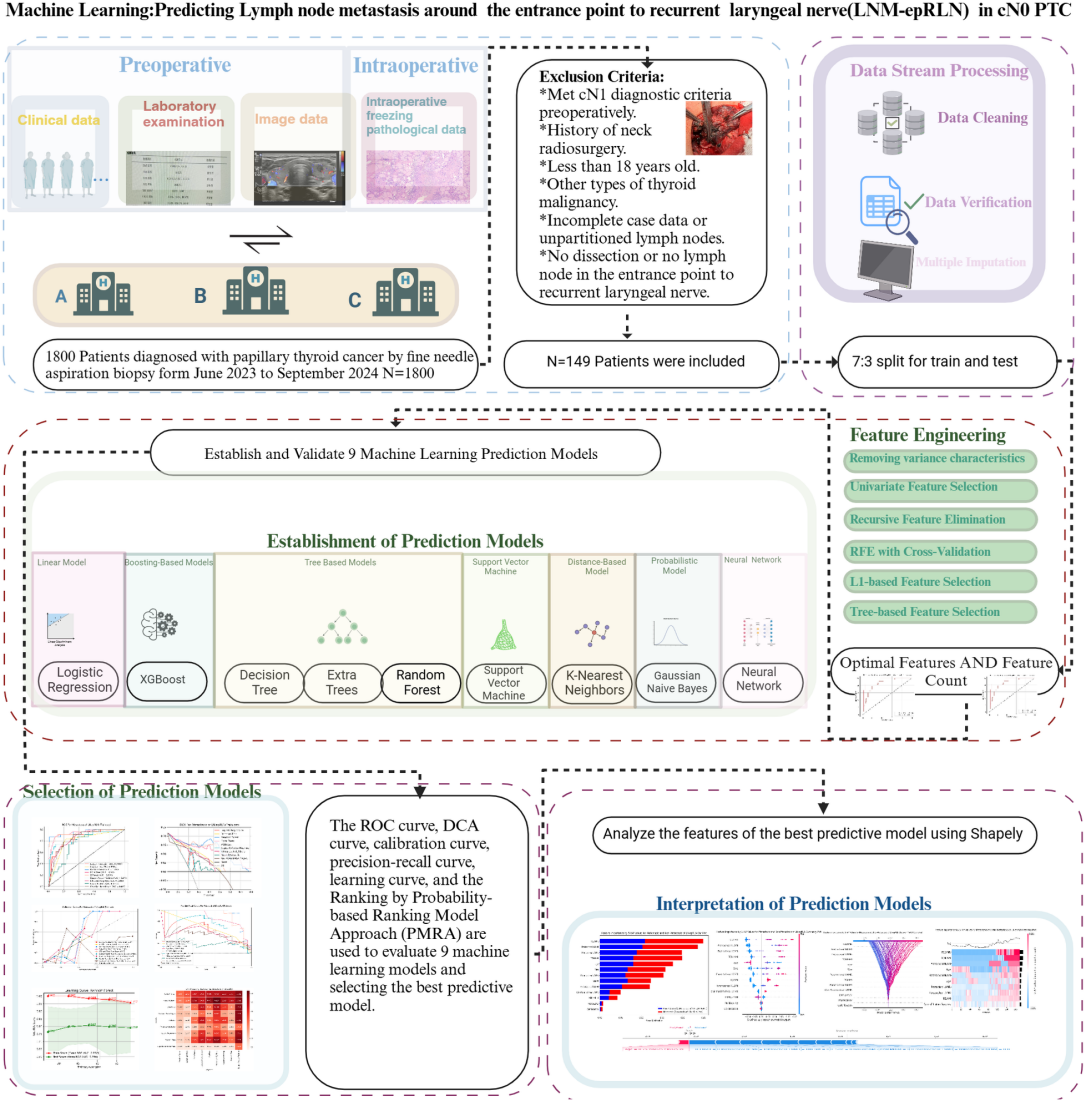
Workflow of the predictive model for lymph node metastasis around the entrance point to recurrent laryngeal nerve (LNM-epRLN) in papillary thyroid carcinoma with clinical node-negative (cN0) classification.

This retrospective study analyzed records from 1800 PTC patients treated between June 2023 and September 2024. The ethics board of the First Affiliated Hospital of Chongqing Medical University approved this study with a waiver of informed consent, as the study was retrospective and deemed to pose minimal risk (Approval number: 2020-181). All methods of this study were performed in accordance with the principles outlined in the Declaration of Helsinki. Inclusion criteria comprised: age >18 years, preoperative fine-needle aspiration confirming PTC, cN0 status, receipt of CLND, and complete clinicopathological data. Exclusion criteria included prior neck surgery or radiotherapy, non-PTC thyroid malignancies, or absence of LN-epRLN dissection. After screening, 149 patients were included and randomly divided into training and testing sets in a 7:3 ratio (**Figure 1**). The data preprocessing included Imputation of missing values using the mode and mean substitution for categorical and continuous variables, respectively. Retention of LNM proportion and count as constant variables due to non-normal distribution. Standardization of numerical features to reduce scale variation and enhance model performance. Use of postoperative histopathology as the gold standard for determining LNM-epRLN. Categorical variables (e.g., sex, tumor border) were encoded using label encoding. Numerical features (e.g., age, tumor size, lymph node counts, and ratios) were standardized to a mean of 0 and a standard deviation of 1 using the StandardScaler from the scikit-learn library to mitigate the influence of differing scales on model performance.

### 2.2 Data collection

Clinical Features (3 variables): Age, sex, and Body Mass Index (BMI). BMI was categorized according to both Chinese and World Health Organization standards: BMI < 18.5 (underweight), 18.5 ≤ BMI < 24 (normal weight), and BMI ≥ 24 (overweight).
Ultrasound Features (9 variables): Parameters included: tumor border (extreme invasion, irregular shape/sharpness, smooth/borderless), calcification (no/large comet tail, coarse calcification, peripheral calcification, microcalcification), tumor internal vascularization, tumor peripheral blood flow, location (non-upper, upper), and aspect ratio, composition, internal echo pattern, internal echo homogeneous.
Intraoperative and Postoperative Features (8 variables): Intraoperative bleeding, Duration of surgery, Length of hospital stay, and Postoperative drainage volume are continuous variables. Postoperative bleeding, RLN injury, hypoparathyroidism, and chyle leak are binary variables.
Pathological Features (8 variables): Tumor size (defined as the largest diameter of the tumor), extrathyroidal extension (ETE) was defined as tumor invasion into tissues beyond the thyroid capsule, including both microscopic and gross (maximum ETE involving the trachea, larynx, and recurrent laryngeal nerve) invasion. ETE was assessed by intraoperative frozen section. Hashimoto’s thyroiditis (HT): Diagnostic criteria included any of the following conditions (1): Thyroid peroxidase antibody level > 50 IU/mL, (2) Ultrasound findings of diffuse heterogeneity, or (3) Histopathological examination showing diffuse lymphocytic thyroiditis(1). Tumor location, concurrent benign tumor, and multifocality were confirmed by ultrasound and intraoperative frozen section. Tumor stage: Determined by intraoperative frozen pathology. BRAF V600E mutation status detected via real-time PCR.
Intraoperative Frozen Pathology Features (20 variables): Total central LNM (TCLNM), prelaryngeal LNM, pretracheal LNM, paratracheal LNM, con-paratracheal LNM, recurrent laryngeal nerve LNM (LNM-prRLN), and lateral LNM (LLNM) are binary variables. The total central lymph node metastasis number (TCNLNM), prelaryngeal NLNM, pretracheal NLNM, paratracheal NLNM, con-paratracheal NLNM, recurrent laryngeal nerve NLNM (NLNM-prRLN), and lateral NLNM are continuous variables. The total central lymph node metastasis ratio (TCLNMR), prelaryngeal LNMR, pretracheal LNMR, paratracheal LNMR, con-paratracheal LNMR, recurrent laryngeal nerve LNMR (LNMR-prRLN), and lateral LNMR are numerical variables that are calculated as the number of metastatic lymph nodes in a region divided by the number of lymph nodes dissected in that region. The dependent variable, which included LNM-epRLN, is a binary variable determined based on the LNM status obtained from the final paraffin-embedded sections.

### 2.3 Surgical method and intraoperative frozen pathology feature extraction

**Figure 2:**
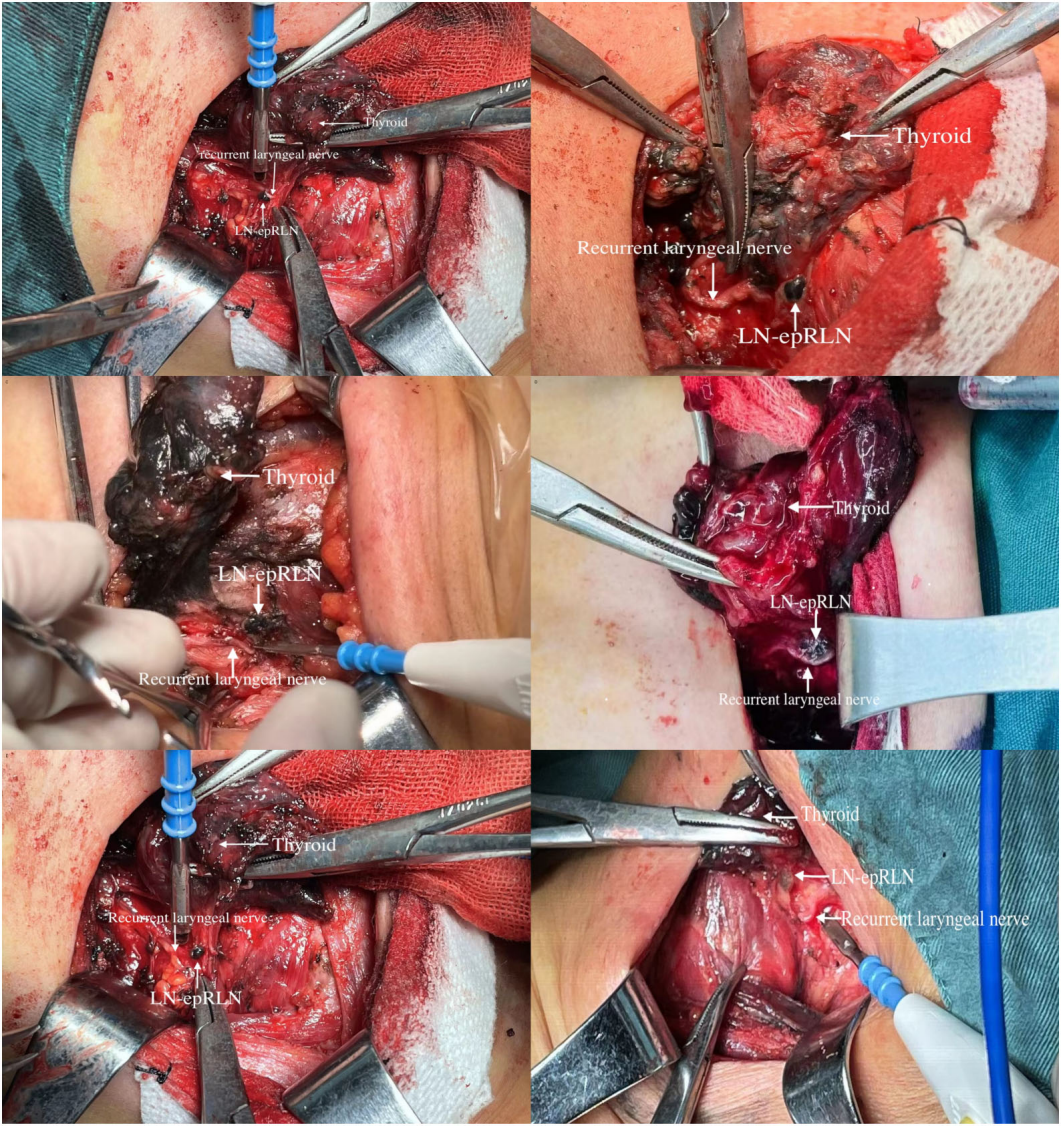
Anatomical localization of lymph nodes around the entrance point of the recurrent laryngeal nerve.

Surgical management consisted of unilateral lobectomy with ipsilateral CLND for tumors <4 cm; total thyroidectomy was performed if intraoperative LNM was detected. Lymph nodes were systematically sampled from prelaryngeal, pretracheal, paratracheal, the entrance point of the RLN, posterior to the RLN, con-paratracheal, and lateral lymph nodes. LN-epRLN was defined as lymphatic tissue within the tracheoesophageal groove surrounding the RLN’s laryngeal entrance point (**Figure 2**). All specimens were labeled and subjected to frozen section analysis intraoperatively, followed by histopathological review by three blinded pathologists. Subsequently, all dissected lymph node specimens, including those from the LN-epRLN region, underwent definitive histopathological review on permanent paraffin-embedded sections. The final pathological diagnosis was established by consensus review of at least two senior pathologists, resolving any initial discrepancies through joint re-examination. The inter-observer agreement for the detection of metastasis in central compartment lymph nodes, assessed on a random subset of 100 cases, was excellent (Cohen’s kappa = 0.89) (27).

### 2.4 Machine learning models

We selected nine supervised machine learning models representing diverse learning paradigms to ensure a comprehensive comparison. This included: a linear model (Logistic Regression, LR) as a simple, interpretable baseline; tree-based models (Decision Tree, DT; Random Forest, RF; Extra Trees, ET; and XGBoost) known for capturing non-linear relationships; distance-based models (Support Vector Machine, SVM; k-Nearest Neighbors, KNN) effective in high-dimensional spaces; a probabilistic model (Gaussian Naive Bayes, GNB) for its efficiency; and a Neural Network (NN) to explore complex hierarchical representations. Model performance was evaluated using accuracy, the area under the receiver operating characteristic curve (AUC), sensitivity, specificity, negative predictive value (NPV), positive predictive value (PPV), F1 score, and false positive rate. All models were implemented using the Python Scikit-learn library. Given the class imbalance in our dataset (with LNM-epRLN being a relatively rare event), we also reported precision-recall (PR) curves, as they provide a more informative assessment of classifier performance under such conditions than ROC curves alone (28).

Hyperparameter tuning was conducted via a grid search strategy combined with 10-fold cross-validation on the training set to optimize model performance and prevent overfitting. The specific hyperparameter search space for each model is detailed in **Supplementary Table S3**.

Dimensionality reduction was performed using six complementary feature selection methods to ensure robustness and identify a stable set of predictive features: variance thresholding (removing low-variance features), univariate selection (filtering based on statistical tests), recursive feature elimination - RFE and RFE-CV (wrapper methods), and L1-based and tree-based selection (embedded methods). This multi-method approach mitigates the bias of any single technique.

### 2.5 SHapley additive explanation analysis

SHAP, a unified, model-agnostic framework from cooperative game theory, was applied to interpret the predictions of the optimal model both globally and locally. This approach quantifies the contribution of each feature to an individual prediction, ensuring explainability independent of the underlying algorithm. Mean absolute SHAP values were used to rank the overall importance of features, and dependence plots were employed to visualize the marginal effect of key variables on the model’s forecasts.

### 2.6 Statistical analysis

All statistical analyses were performed using R software (version 4.3.2; R Foundation for Statistical Computing). Categorical variables are presented as frequencies and percentages, while continuous variables are summarized as either the mean ± standard deviation or the median with interquartile range, depending on their distribution. Group comparisons for categorical variables were conducted using the Chi-square test or Fisher’s exact test, as appropriate. Machine learning workflows, including feature selection, model training, hyperparameter tuning, and evaluation, were implemented in Python (version 3.11.5). The scikit-learn library (version 0.24) was used to develop all models and compute performance metrics. The code used for both statistical and machine learning analyses is publicly available on GitHub (https://github.com/ZJ573693/ML-for-LN-epRLN).

## 3 Results

### 3.1 Clinical and ultrasonographic pathological characteristics of the patients

A total of 1,800 cN0-PTC patients were initially enrolled, among whom 149 patients (8.3%) were found to have identifiable LN-epRLN tissue upon dissection. The metastasis rate of LNM-epRLN was 19.46% (29/149). The cohort of these 149 patients consisted of 115 females and 34 males (female-to-male ratio 3.38:1), with a mean age of 43.06 ± 12.120 years. Univariate analysis revealed significant associations between LNM-epRLN and several clinicopathological factors (**Table 1**). Patients with LNM-epRLN had larger tumor sizes (15.81 ± 9.29 mm vs. 9.68 ± 6.18 mm, *P* < 0.001), were more frequently aged ≤45 years (68.97% vs. 31.03%, *P* = 0.007), and had greater postoperative drainage volumes (74.14 ± 71.34 mL vs. 48.13 ± 31.79 mL, *P* = 0.003) compared to those without metastasis. LNM-epRLN was significantly correlated with metastasis in multiple lymph node regions, including TCLNM (96.55% vs. 3.45%, *P* < 0.001), LLNM (72.41% vs. 10.34%, *P* < 0.001), and contralateral paratracheal LNM (31.03% vs. 24.14%, *P* < 0.001). Furthermore, significant associations were observed with metastasis in the prelaryngeal, pretracheal, and ipsilateral paratracheal lymph nodes (all *P* < 0.001), as well as with LNM-prRLN (*P* = 0.001). The number and ratio of metastatic lymph nodes within the central and lateral compartments were also significantly higher in the LNM-epRLN group (all *P* < 0.05).

**Table 1:** Clinical features, ultrasound image features, intraoperative frozen section pathology features, and univariate analysis of risk factors for LNM-epRLN.

Characteristics	Total(N = 149)		*p*-value
	LNM-epRLN (+)	LNM-epRLN (–)	
Age Mean ± SD	37.690 ± 10.651	44.425 ± 12.157	0.007
≤45	20 (68.966%)	67 (55.833%)	
>45	9 (31.034%)	53 (44.167%)	
Sex			0.155
female	19 (65.517%)	96 (80.000%)	
male	10 (34.483%)	24 (20.000%)	
BMI			0.665
normal	16 (55.172%)	66 (55.000%)	
overweight	11 (37.931%)	50 (41.667%)	
underweight	2 (6.897%)	4 (3.333%)	
Tumor border			0.389
irregular shape or sharpness	27 (93.103%)	107 (89.167%)	
extrandular invasion	0(0.00%)	7 (5.833%)	
smooth or borderless	2 (6.897%)	6 (5.000%)	
Aspect ratio			0.576
>1	26 (89.655%)	100 (83.333%)	
≤1	3 (10.345%)	20 (16.667%)	
Composition			0.727
solid	27 (93.103%)	116 (96.667%)	
Non-solid	2 (6.897%)	4 (3.333%)	
Internal echo pattern			0.756
hypoechoic	26 (89.655%)	112 (93.333%)	
high/isoechoic	2 (6.897%)	6 (5.000%)	
very hypoechoic	1 (3.448%)	2 (1.667%)	
Internal echo homogeneous			0.619
Non-Uniform	24 (82.759%)	106 (88.333%)	
Uniform	5 (17.241%)	14 (11.667%)	
Calcification			0.061
Microcalcification	19 (65.517%)	83 (69.167%)	
no or large comet tail	6 (20.690%)	31 (25.833%)	
peripheral calcification	4 (13.793%)	3 (2.500%)	
coarse calcification	0 (0.00%)	3 (2.500%)	
Tumor internal vascularization			1
Abundant	21 (72.414%)	87 (72.500%)	
Without	8 (27.586%)	33 (27.500%)	
Tumor Peripheral blood flow			0.812
Abundant	21 (72.414%)	92 (76.667%)	
Without	8 (27.586%)	28 (23.333%)	
Location			0.222
Non-Upper	27 (93.103%)	98 (81.667%)	
Upper	2 (6.897%)	22 (18.333%)	
Size			
Mean ± SD	15.807 ± 9.288	9.682 ± 6.178	<0.001
BRAF			0.376
Yes	25 (86.207%)	112 (93.333%)	
No	4 (13.793%)	8 (6.667%)	
Concurrent benign tumor			0.51
No	26 (89.655%)	99 (82.500%)	
Yes	3 (10.345%)	21 (17.500%)	
Mulifocality			0.549
No	16 (55.172%)	76 (63.333%)	
Yes	13 (44.828%)	44 (36.667%)	
Hashimoto’s thyroiditis			0.824
No	22 (75.862%)	86 (71.667%)	
Yes	7 (24.138%)	34 (28.333%)	
mETE			0.438
No	24 (82.759%)	108 (90.000%)	
Yes	5 (17.241%)	12 (10.000%)	
gETE			0.399
No	29 (100.000%)	113 (94.167%)	
Yes	0(0.00%)	7 (5.833%)	
Tumor stage			0.652
1	24 (82.759%)	104 (86.667%)	
2	2 (6.897%)	6 (5.000%)	
3	3 (10.345%)	7 (5.833%)	
4	0(0.00%)	3 (2.500%)	
Intraoperative bleeding			
Mean ± SD	21.207 ± 11.776	17.958 ± 19.212	0.385
Duration of surgery			
Mean ± SD	113.414 ± 38.277	108.008 ± 42.476	0.532
Length of hospital stay			
Mean ± SD	3.759 ± 1.618	3.600 ± 1.170	0.546
Postoperative drainage volume			
Mean ± SD	74.138 ± 71.340	48.125 ± 31.789	0.003
Postoperative bleeding			1
No	27 (93.103%)	114 (95.000%)	
Yes	2 (6.897%)	6 (5.000%)	
Recurrent laryngeal nerve injury			1
No	29 (100.000%)	120 (100.000%)	
Yes	0(0.00%)	0(0.00%)	
Hypoparathyroidism			0.329
No	23 (79.310%)	106 (88.333%)	
Yes	6 (20.690%)	14 (11.667%)	
Chyle leak			1
No	28 (96.552%)	117 (97.500%)	
Yes	1 (3.448%)	3 (2.500%)	
Prelaryngeal LNMR			
Mean ± SD	0.363 ± 0.433	0.091 ± 0.244	<0.001
Prelaryngeal NLNM			
Mean ± SD	0.724 ± 1.032	0.233 ± 0.807	0.006
Prelaryngeal LNM			<0.001
No	12 (41.379%)	85 (70.833%)	
Yes	13 (44.828%)	14 (11.667%)	
	4 (13.793%)	21 (17.500%)	
Pretracheal LNMR			
Mean ± SD	0.423 ± 0.355	0.129 ± 0.263	<0.001
Pretracheal NLNM			
Mean ± SD	1.897 ± 1.915	0.617 ± 1.291	<0.001
Pretracheal LNM			<0.001
No	9 (31.034%)	88 (73.333%)	
Yes	20 (68.966%)	32 (26.667%)	
Paratracheal LNMR			
Mean ± SD	0.460 ± 0.350	0.158 ± 0.273	<0.001
Paratracheal NLNM			
Mean ± SD	2.276 ± 2.136	0.742 ± 1.325	<0.001
Paratracheal LNM			<0.001
No	7 (24.138%)	78 (65.000%)	
Yes	22 (75.862%)	42 (35.000%)	
Con-Paratracheal LNMR			
Mean ± SD	0.390 ± 0.398	0.086 ± 0.182	0.001
Con-Paratracheal NLNM			
Mean ± SD	1.500 ± 1.673	0.324 ± 0.727	0.001
Con-Paratracheal LNM			<0.001
No	7 (24.138%)	27 (22.500%)	
Yes	9 (31.034%)	7 (5.833%)	
	13 (44.828%)	86 (71.667%)	
LNMR-prRLN			
Mean ± SD	0.342 ± 0.401	0.043 ± 0.153	0.001
NLNM-prRLN			
Mean ± SD	0.733 ± 0.884	0.109 ± 0.315	0.001
LNM-prRLN			0.001
No	7 (24.138%)	56 (46.667%)	
Yes	8 (27.586%)	57 (47.500%)	
	14 (48.276%)	7 (5.833%)	
TCLNMR			
Mean ± SD	0.413 ± 0.228	0.128 ± 0.194	<0.001
TCNLNM			
Mean ± SD	6.103 ± 4.143	1.742 ± 2.851	<0.001
TCLNM			<0.001
No	1 (3.448%)	65 (54.167%)	
Yes	28 (96.552%)	55 (45.833%)	
LLNMR			
Mean ± SD	0.285 ± 0.246	0.077 ± 0.104	<0.001
LNLNM			
Mean ± SD	5.292 ± 4.648	1.962 ± 3.592	0.001
LLNM			<0.001
No	3 (10.345%)	29 (24.167%)	
Yes	21 (72.414%)	23 (19.167%)	
	5 (17.241%)	68 (56.667%)	

LNM-epRLN, lymph node metastasis around the entrance point to the recurrent laryngeal nerve; BMI, body mass index; BRAF, V-raf murine sarcoma viral oncogene homolog B1; mETE, minimal extrathyroidal extension; gETE, gross extrathyroidal extension; LNM, lymph node metastasis; LNMR, lymph node metastasis ratio; SD, standard deviation; NLNM, number of lymph node metastasis; LNM-prRLN, lymph node metastasis posterior to the right recurrent laryngeal nerve; TCLNMR, ratio of total central lymph node metastasis; TCNLNM, number of total central lymph node metastasis; TCLNM, total central lymph node metastasis; LLNMR, ratio of lateral neck lymph node metastasis; LNLNM, number of lateral neck lymph node metastasis; LLNM, lateral neck lymph node metastasis.

### 3.2 Development and assessment of machine learning prediction models

Forty-eight feature variables were initially included. Dimensionality reduction was performed using six feature selection methods: variance thresholding, univariate selection, recursive feature elimination (RFE), cross-validated RFE (RFE-CV), L1-based selection, and tree-based selection (**Supplementary Figures 1A1-F1**). Through L1-regularized feature selection, 13 predictive variables were identified for predicting LNM-epRLN, including multifocality, calcification, con-paratracheal LNMR, LNM-prRLN, TCLNM, pretracheal LNMR, pretracheal NLNM, paratracheal NLNM, TCLNMR, TCNLNM, tumor size, age, and paratracheal LNMR (see **Supplementary Table S2**).

**Figure 3:**
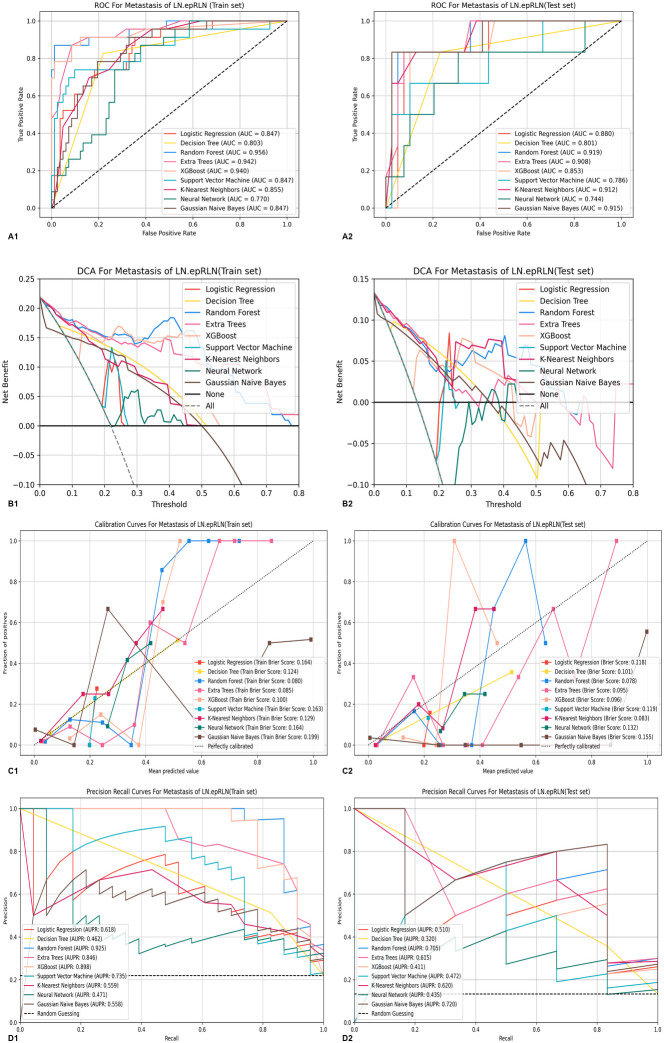
**(A)** ROC curves of the 9 machine learning predictive models on the training, test sets: **(A1)** ROC curve on the training set, **(A2)** ROC curve on the validation set. **(B)** DCA curves of the 9 machine learning predictive models on the training and test sets: **(B1)** DCA curve on the training set, **(B2)** DCA curve on the validation set. **(C)** Calibration curves of the 9 machine learning predictive models on the training and test sets: **(C1)** Calibration curve on the training set, **(C2)** Calibration curve on the validation set. **(D)** Precision-recall curves on the 9 machine learning predictive models on the training and test sets: **(D1)** Precision-recall curve on the training set, **(D2)** Precision-recall curve on the validation set. ROC, Receiver Operating Characteristic; DCA, Decision curve analysis; LN-epRLN, Lymph Nodes around the entrance point of Recurrent Laryngeal Nerve.

These features were used to train nine ML models: LR, DT, RF, ET, XGBoost, SVM, KNN, NN, and GNB. Hyperparameter tuning was conducted using tenfold cross-validation and grid search. Model performance was evaluated using accuracy, AUC, sensitivity, and specificity as primary metrics, with seven additional indicators supporting these metrics (see **Supplementary Table S1**). Evaluation plots included ROC, decision curve analysis (DCA), calibration, precision–recall, and learning curves (**Figures 3A–D**).

**Figure 4:**
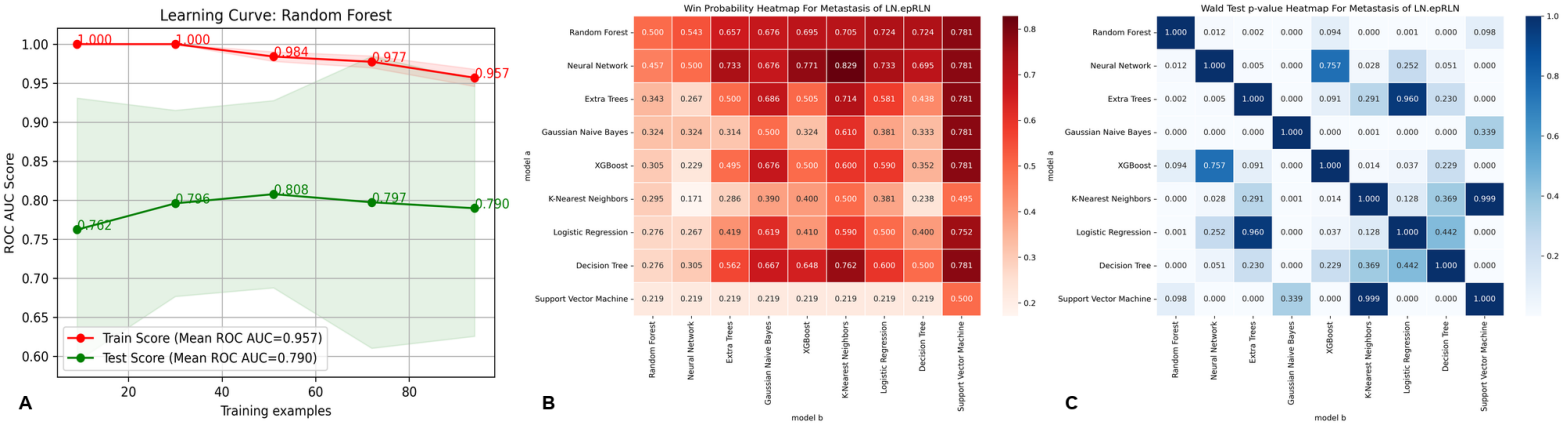
Comparison of learning curves and win probabilities estimated by PMRA and wald test p-values for the best machine learning model-random forest in predicting metastasis of LN-epRLN. **(A)** Learning curve of Random Forest for Metastasis of LN-epRLN; **(B)** Win probabilities estimated by PMRA for predicting Metastasis of LN-epRLN; **(C)** Wald test p-values for predicting Metastasis of LN-epRLN. PMRA, Probability-based Ranking Model Approach; LN-epRLN: Lymph Nodes around entrance point of the Recurrent Laryngeal Nerve.

The RF model demonstrated the highest predictive performance, with training and testing AUC values of 0.956 and 0.919, respectively (**Supplementary Table S1**, **Supplementary Figures 1E1-E3**). DCA indicated that the RF model provided superior clinical utility across most probability thresholds (**Figures 3B1-B2**). The learning curve suggested stable generalization despite slight overfitting (**Figure 4A**). Based on probability-based ranking analysis (PMRA), RF had the lowest overfitting risk and optimal predictive value among all nine ML models (Win Probability < 0.5) (**Figure 4B**). Wald tests confirmed that RF’s advantage was statistically significant compared to most models (*P* < 0.05), though not against XGBoost (**Figure 4C**). Calibration and precision–recall curves further corroborated RF’s robustness.

### 3.3 Model interpretability and feature importance based on SHAP analysis

**Figure 5:**
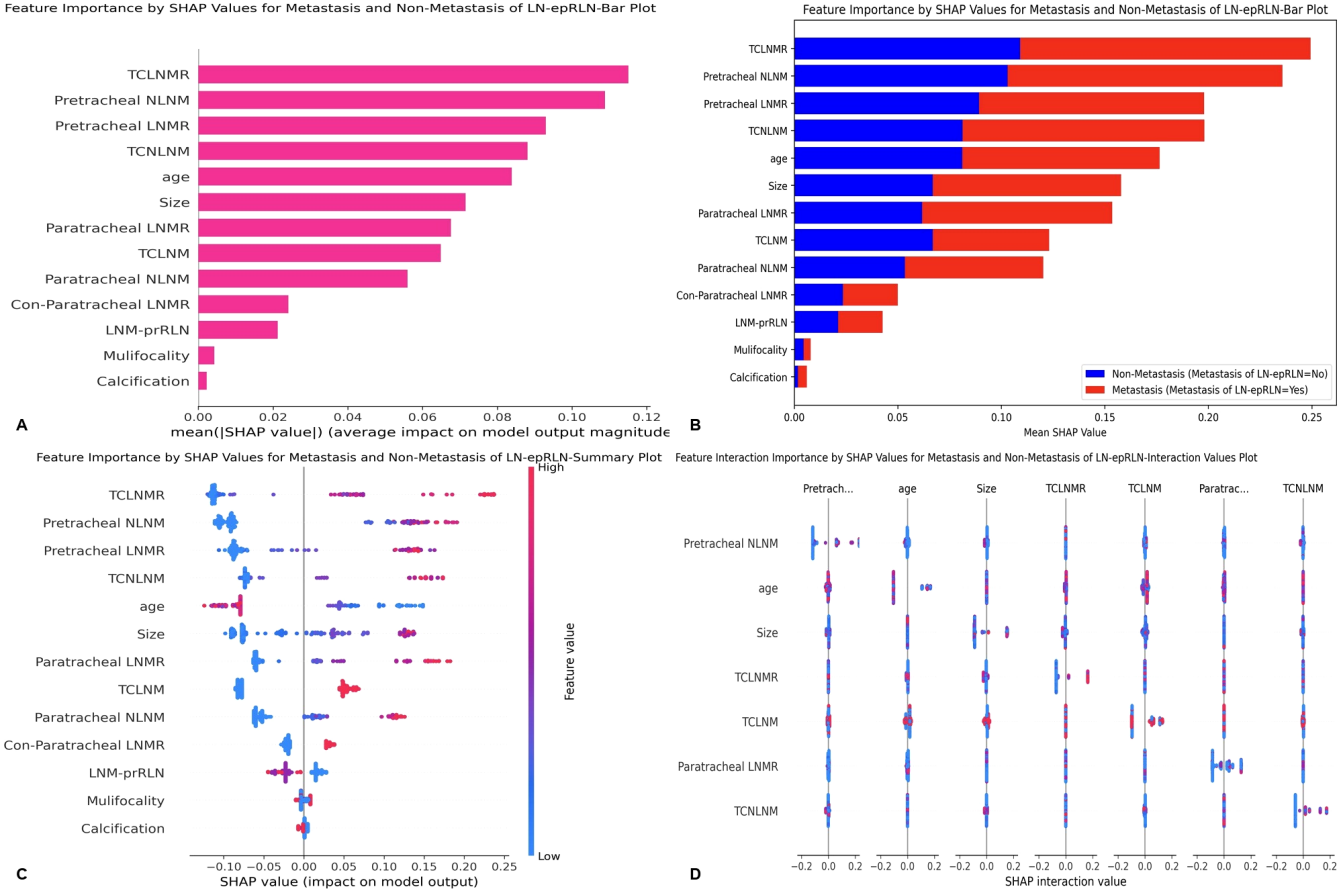
SHAP analysis of the random forest model: **(A)** Standard bar chart of SHAP summary plot, showing the impact of each feature on the Random Forest model: **(B)** Categorical bar chart of SHAP summary plot, also showing the impact of each feature; **(C)** SHAP summary scatter plot, visually reflecting the relationship between feature values and predicted probabilities; **(D)** SHAP interaction plot for the top 7 features affecting the prediction of Metastasis of LN-epRLN. SHAP, Shapley Additive Explanations; LN-epRLN, Lymph nodes around the entrance point of Recurrent Laryngeal Nerve; TCLNMR, Ratio of Total Central Lymph Node Metastasis; NLNM, Number of Lymph Node Metastasis; LNMR, Ratio of Lymph Node Metastasis; TCNLNM, Number of Total Central Lymph Node Metastasis; TCLNM, Total Central Lymph Node Metastasis; LNM-prRLN, Lymph Node Metastasis posterior to the right Recurrent Laryngeal Nerve.

**Figure 6:**
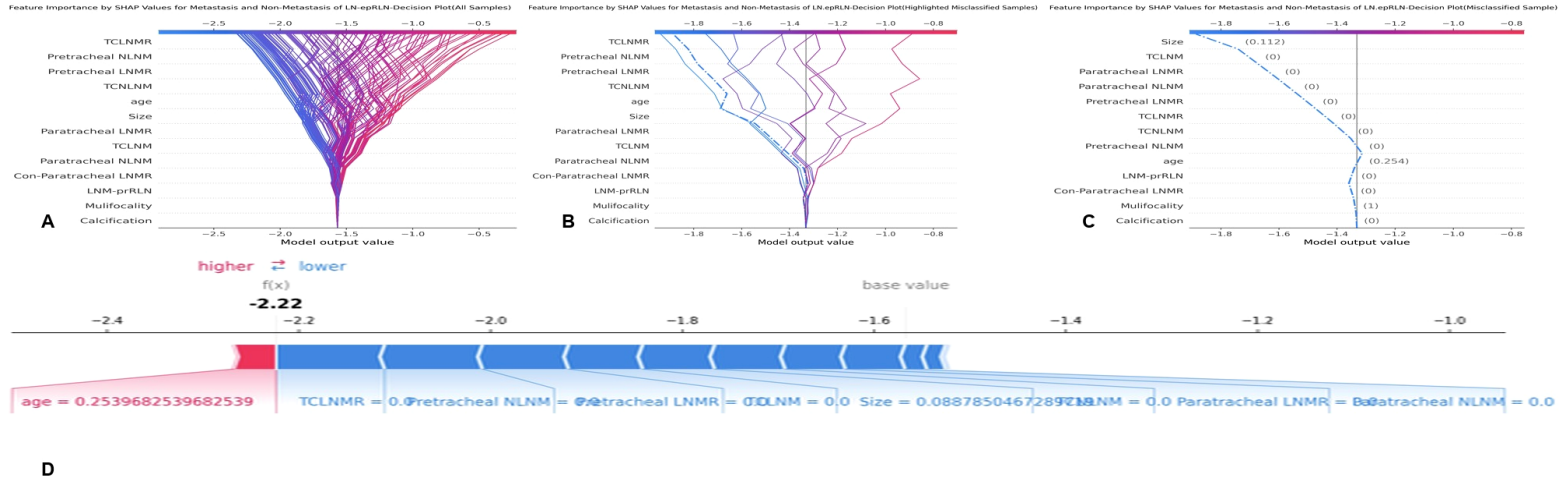
**(A)** SHAP decision plot for all PTC patients; **(B)** SHAP decision plot for 20 randomly selected PTC patients, with one misclassified case (shown by the dashed line); **(C)** Detailed SHAP decision plot for the misclassified case. **(D)** Force plot of single case. SHAP, Shapley Additive Explanations; PTC, Papillary Thyroid Carcinoma; LN-epRLN, Lymph nodes around the entrance point of Recurrent Laryngeal Nerve; TCLNMR, Ratio of Total Central Lymph Node Metastasis; NLNM, Number of Lymph Node Metastasis; LNMR, Ratio of Lymph Node Metastasis: TCNLNM, Number of Total Central Lymph Node Metastasis; TCLNM, Total Central Lymph Node Metastasis; LNM-prRLN, Lymph Node Metastasis posterior to the right Recurrent Laryngeal Nerve.

SHAP analysis was employed to interpret the RF model and quantify the contributions of each feature. The feature importance ranking, based on mean absolute SHAP values, identified the following as the top ten predictors of LNM-epRLN: TCLNMR, pretracheal NLNM, pretracheal LNMR, TCNLNM, age, tumor size, paratracheal LNMR, TCLNM, paratracheal NLNM, and con-paratracheal LNMR (**Figure 5A**). The bar chart displays the distribution of LNM cases (red) and non-metastasis cases (blue) (**Figure 5B**). The SHAP summary plot (**Figure 5C**) illustrated the directional relationship between feature values and prediction probability. Higher values of TCLNMR, pretracheal NLNM, pretracheal LNMR, TCNLNM, age, and tumor size were consistently associated with an increased risk of LNM-epRLN. Interaction effects between features were further visualized using a SHAP interaction plot (**Figure 5D**). Decision plots (**Figure 6**) illustrated the individual contributions of each feature to the final prediction, highlighting the sample-specific reasoning process underlying the model’s output. Each line represents a patient, with SHAP values indicating the magnitude and direction of each feature’s influence.

**Figure 7:**
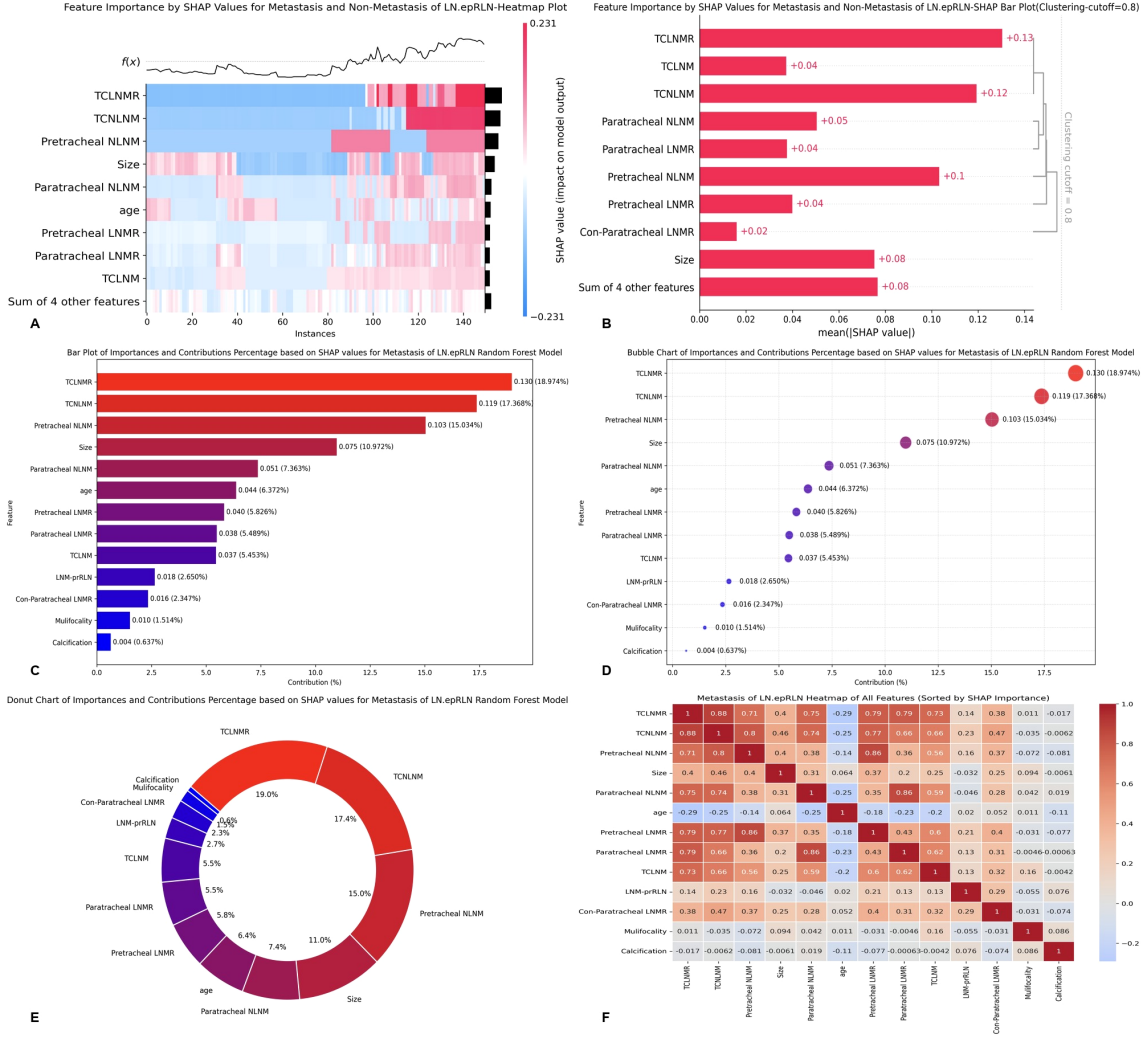
**(A)** SHAP absolute value heatmap for all PTC patients; **(B)** Bar chart of SHAP mean absolute values with clustering analysis for PTC patients; **(C)** Bar chart showing the contribution percentage of the feature variables based on SHAP values; **(D)** Bubble plot showing the contribution percentage of the feature variables based on SHAP; **(E)** Donut chart showing the contribution percentage of the feature variables based on SHAP; **(F)** Heatmap plot showing the contribution percentage of the feature variables based on SHAP. SHAP, Shapley Additive Explanations; LN-epRLN, Lymph nodes around the entrance point of Recurrent Laryngeal Nerve; TCLNMR, Ratio of Total Central Lymph Node Metastasis; TCNLNM, Number of Total Central Lymph Node Metastasis; NLNM, Number of Lymph Node Metastasis; LNMR, Ratio of Lymph Node Metastasis; TCLNM, Total Central Lymph Node Metastasis; LNM-prRLN, Lymph Node Metastasis posterior to the right Recurrent Laryngeal Nerve.

Quantitative assessment of feature contributions revealed that TCLNMR was the most influential predictor (SHAP importance: 0.130, contribution: 18.97%), followed by TCNLNM (0.119, 17.37%), pretracheal NLNM (0.103, 15.03%), tumor size (0.075, 10.97%), and paratracheal NLNM (0.051, 7.36%) (**Figures 7A, B**). The combined contributions of these features accounted for the majority of the model’s predictive output (**Figures 7C–F**).

## 4 Discussion

To the best of our knowledge, this is the first study to develop and validate an ML model specifically for predicting LNM-epRLN in patients with cN0-PTC. The RF algorithm demonstrated strong predictive performance, with an AUC of 0.919. SHAP analysis identified central lymph node metastasis burden, particularly the TCLNMR, as the most influential predictor, shifting LNM-epRLN risk assessment from subjective judgment to a quantitative and interpretable framework.

Although the clinical relevance of the LN-epRLN subregion has been historically overlooked, earlier work by Lu Tian et al. (29, 30) helped define this area and reported metastasis rates ranging from 2.51% to 3.76% (17, 21). Our findings confirm central LNM as a key risk factor and further quantify its dominant role via SHAP, where TCLNMR was the most significant contributor (26, 31). This underscores that the overall tumor burden in the central compartment, rather than isolated nodal metastasis, is the primary driver of LNM-epRLN. This observation is consistent with prior reports indicating that over 93% of LN-epRLN-positive patients exhibit concurrent central LNM (18, 29, 30).

A central challenge in thyroid surgery is balancing thorough dissection against procedural risks. LN-epRLN dissection is anatomically demanding, with proximity to the RLN and parathyroid glands raising concerns for complications (32–34). However, leaving behind metastatic nodes increases the risk of recurrence and complicates future reoperations (35, 36). Conventional decision-making relies heavily on the surgeon’s experience (37). Enhanced by SHAP interpretability, our RF model provides an individualized, visual assessment of LNM-epRLN risk. This aligns with the goal of predictive approaches to treatment effect heterogeneity, which advocate for using models to guide individualized therapy by identifying patients who are most likely to benefit from a specific intervention (38). It thereby empowers surgeons to tailor the extent of dissection, being more aggressive in high-risk cases and more conservative in low-risk ones, and optimizes the risk-benefit balance in line with precision surgery.

Recent multicenter studies have successfully developed ML models for predicting lateral lymph node metastasis in PTC (9, 36), demonstrating the feasibility and value of such approaches. Meanwhile, other studies have highlighted the potential of integrating multimodal data; for instance, a model combining deep learning, radiomics, and clinical features showed superior performance in diagnosing central lymph node metastasis (31). Our study contributes a focused model for the specific and surgically challenging LN-epRLN subregion. As highlighted by the TRIPOD-AI guidelines (39), transparent reporting and external validation are the next steps for translation. The logical progression for this work is, therefore, external validation across multiple institutions to assess its generalizability, akin to the path taken by these earlier models (36).

The clinical translation of a predictive model depends not only on its accuracy but also on the safety of the procedure it guides and the transparency of its decision-making process (40, 41). In our cohort, compared to patients without dissection, the dissection group showed no significant differences in operative time, hospitalization duration, bleeding, nerve injury, or chyle leak (**Table 1**). Although postoperative drainage volume was higher in the dissection group (*P* = 0.03), likely reflecting more extensive tissue removal, this did not translate into an increased clinical risk of bleeding. These findings provide preliminary support for the safety of a model-guided approach. Furthermore, to enhance technical precision, we employed complementary intraoperative techniques. First, carbon nanoparticles were used for lymphatic mapping (42–44), aiding in parathyroid preservation and lymph node visualization. In the confined anatomical space of the LN-epRLN, this technique helps distinguish lymphatic tissue from neural and glandular structures. Second, in alignment with international standards (45), intraoperative neuromonitoring (IONM) of the RLN was routinely employed. This practice provides real-time functional feedback, aids in the identification of the nerve within the surgical field, and is instrumental in preventing iatrogenic injury during dissection around the critical LN-epRLN region. The combined use of these techniques is intended to maximize the safety of performing a precise dissection in this high-risk area.

Notably, we developed a simplified model incorporating only nine key predictors, which significantly enhances clinical practicality while retaining high predictive accuracy (36). Most of these features can be obtained through preoperative ultrasound evaluation and intraoperative frozen section analysis, making the real-time application of the model feasible (46). SHAP decision plots and force plots further enhance model transparency by illustrating the contribution of each feature to the final prediction for individual patients, effectively transforming a “black box” model into an interpretable “glass box” (26, 47). This transparency is crucial for building clinical trust and facilitating the adoption of the model among surgeons.

This study has several limitations. First, its single-center, retrospective design is subject to inherent selection bias and may limit the generalizability of our findings. The model was developed and validated on data from a single institution with specific surgical and pathological protocols. Second, although our cohort is sizable, LNM-epRLN remains a relatively rare event, resulting in a limited number of positive cases (n=29) for model training. This class imbalance, while reflective of the clinical reality of this specific subregion, likely contributes to the model’s moderate sensitivity and underscores the need for larger, pooled datasets in future work. Finally, the model’s sensitivity of 0.500 in the test set indicates room for improvement, suggesting that future work should aim to identify additional predictive features or employ techniques to address class imbalance.

## 5 Conclusion

In this study, we successfully developed and validated an interpretable ML framework for predicting LNM-epRLN in patients with cN0 PTC. The RF model demonstrated superior performance, achieving a high AUC of 0.919. Through SHAP analysis, we identified and quantified the importance of key predictors, with the TCLNMR being the most influential factor. A simplified model utilizing only seven clinically accessible features retained robust predictive power, enhancing its potential for practical integration into the surgical workflow.

This interpretable model provides a data-driven tool that can assist surgeons in making individualized, intraoperative decisions regarding the dissection of the high-risk LN-epRLN region. By offering a quantitative assessment of metastatic risk, it aims to optimize the critical balance between oncological completeness and the preservation of RLN function. Future efforts must prioritize external validation in multi-center, prospective cohorts to rigorously assess the model’s generalizability and clinical utility across different practice settings. Following successful validation, the incorporation of additional biomarkers may further refine predictive accuracy, with the ultimate goal of an AI-assisted decision system for advancing precision surgery in thyroid cancer.

## Data Availability

The raw data supporting the conclusions of this article will be made available by the authors, without undue reservation.

## Supplementary material

Supplementary Figure 1 ROC Curves of Variables Selected by Six Machine Learning Methods in Nine Machine Learning Algorithms and the ROC and DCA Curves of the Optimal Prediction Model in Train and Test Sets for Lymph Node Metastasis around the entrance point to Recurrent Laryngeal Nerve (LNM-epRLN): **(A)**, Removing variance characteristics. **(B)**, Univariate Feature Selection. **(C)**, Recursive Feature Elimination (RFE). **(D)**, RFE with Cross-Validation (RFE-CV), **(E)**. L1-Based Feature Selection, **(F)**. Tree-based Feature Selection.
