# Supplementary figures and images for: Machine learning: predicting lymph node metastasis around the entrance point to the recurrent laryngeal nerve in cN0 papillary thyroid carcinoma
Source: Front Endocrinol (Lausanne). 2026 Mar 2;17:1721148. doi: 10.3389/fendo.2026.1721148 (PMC12989384; doi:10.3389/fendo.2026.1721148)

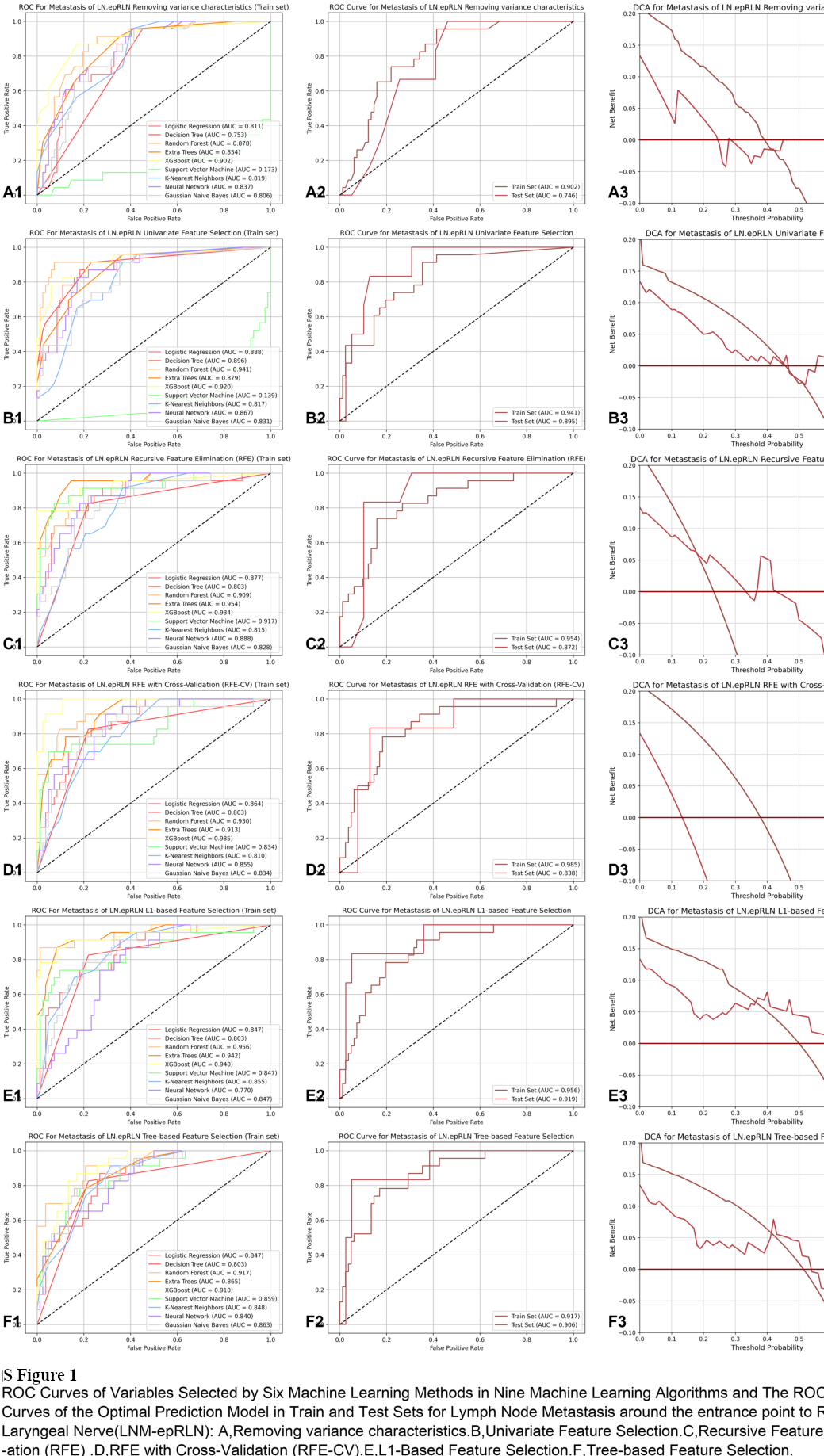

Supplement: Supplementary Figure 1 — ROC Curves of Variables Selected by Six Machine Learning Methods in Nine Machine Learning Algorithms and the ROC and DCA Curves of the Optimal Prediction Model in Train and Test Sets for Lymph Node Metastasis around the entrance point to Recurrent Laryngeal Nerve (LNM-epRLN): (A), Removing variance characteristics. (B), Univariate Feature Selection. (C), Recursive Feature Elimination (RFE). (D), RFE with Cross-Validation (RFE-CV), (E). L1-Based Feature Selection, (F). Tree-based Feature Selection. [file Image1.tiff]
