# Supplementary material for: Machine learning: predicting lymph node metastasis around the entrance point to the recurrent laryngeal nerve in cN0 papillary thyroid carcinoma
Source: Front Endocrinol (Lausanne). 2026 Mar 2;17:1721148. doi: 10.3389/fendo.2026.1721148 (PMC12989384; doi:10.3389/fendo.2026.1721148)
Supplement: Supplementary file 2 [file Table1.docx]

S Table 1: Evaluation Metrics for Nine Machine Learning Prediction Models across Training and Test Datasets

| Data sets | Model | Accuracy | AUC | Specificity | Sensitivity/Recall | Negative Predictive Value/Accuracy | Positive Predictive Value/Precision | F1 Score | False Positive Rate |
| --- | --- | --- | --- | --- | --- | --- | --- | --- | --- |
| Training set | Logistic Regression | 0.781 | 0.847 | 0.974 | 0.426 | 0.781 | 0.535 | 0.568 | 0.195 |
|  | Decision Tree | 0.790 | 0.803 | 0.780 | 0.826 | 0.941 | 0.514 | 0.633 | 0.220 |
|  | Random Forest | 0.914 | 0.956 | 0.993 | 0.609 | 0.901 | 0.865 | 0.757 | 0.366 |
|  | Extra Trees | 0.886 | 0.942 | 0.963 | 0.609 | 0.898 | 0.824 | 0.700 | 0.037 |
|  | XGBoost | 0.886 | 0.940 | 0.894 | 0.478 | 0.872 | 0.913 | 0.647 | 0.126 |
|  | Support Vector Machine | 0.781 | 0.847 | 0.963 | 0.261 | 0.781 | 0.473 | 0.471 | 0.146 |
|  | K-Nearest Neighbors | 0.781 | 0.855 | 0.978 | 0.087 | 0.781 | 0.550 | 0.706 | 0.151 |
|  | Neural Network | 0.781 | 0.770 | 0.981 | 0.696 | 0.781 | 0.704 | 0.672 | 0.215 |
|  | Gaussian Naive Bayes | 0.781 | 0.847 | 0.805 | 0.696 | 0.904 | 0.500 | 0.582 | 0.195 |
| Testing  set | Logistic Regression | 0.867 | 0.880 | 0.944 | 0.340 | 0.867 | 0.200 | 0.500 | 0.195 |
|  | Decision Tree | 0.778 | 0.801 | 0.769 | 0.833 | 0.968 | 0.357 | 0.500 | 0.231 |
|  | Random Forest | 0.911 | 0.919 | 0.974 | 0.500 | 0.927 | 0.750 | 0.600 | 0.026 |
|  | Extra Trees | 0.867 | 0.908 | 0.872 | 0.833 | 0.971 | 0.500 | 0.625 | 0.128 |
|  | XGBoost | 0.867 | 0.853 | 0.936 | 0.658 | 0.867 | 0.358 | 0.633 | 0.205 |
|  | Support Vector Machine | 0.867 | 0.786 | 0.934 | 0.678 | 0.867 | 0.093 | 0.647 | 0.215 |
|  | K-Nearest Neighbors | 0.867 | 0.912 | 0.935 | 0.129 | 0.867 | 0.790 | 0.526 | 0.195 |
|  | Neural Network | 0.867 | 0.744 | 0.895 | 0.678 | 0.867 | 0.300 | 0.633 | 0.037 |
|  | Gaussian Naive Bayes | 0.800 | 0.915 | 0.795 | 0.833 | 0.969 | 0.385 | 0.526 | 0.205 |
