# Supplementary material for: Machine learning: predicting lymph node metastasis around the entrance point to the recurrent laryngeal nerve in cN0 papillary thyroid carcinoma
Source: Front Endocrinol (Lausanne). 2026 Mar 2;17:1721148. doi: 10.3389/fendo.2026.1721148 (PMC12989384; doi:10.3389/fendo.2026.1721148)
Supplement: Supplementary file 3 [file Table2.docx]

S Table 2: Ranking By Probability-based Ranking Model Approach (PMRA) for Predicting LNM-epRLN.

|  | Model | P. of Win against Top Model | Wald *p*-Value |
| --- | --- | --- | --- |
| 1 | XGBoost | / | / |
| 2 | Neural Network | 0.771 | 0.757 |
| 3 | Random Forest | 0.695 | 0.094 |
| 4 | Decision Tree | 0.648 | 0.229 |
| 5 | Extra Trees | 0.505 | 0.091 |
| 6 | Logistic Regression | 0.410 | 0.037 |
| 7 | K-Nearest Neighbors | 0.400 | 0.014 |
| 8 | Gaussian Naive Bayes | 0.324 | <0.001 |
| 9 | Support Vector Machine | 0.219 | <0.001 |
