# Supplementary material for: Machine learning: predicting lymph node metastasis around the entrance point to the recurrent laryngeal nerve in cN0 papillary thyroid carcinoma
Source: Front Endocrinol (Lausanne). 2026 Mar 2;17:1721148. doi: 10.3389/fendo.2026.1721148 (PMC12989384; doi:10.3389/fendo.2026.1721148)
Supplement: Supplementary file 4 [file Table3.docx]

S Table 3. Hyperparameter search spaces for the nine machine learning models. All models were tuned using the same preprocessing pipeline and a comparable grid-search budget as described below.

| Model | Hyperparameter | Search Space / Values Tried |
| --- | --- | --- |
| Logistic Regression | C | [0.01, 1, 100] |
|  | solver | ['lbfgs', 'liblinear', 'saga'] |
|  | max_iter | [200, 500, 1000] |
|  | class_weight | [None, 'balanced'] |
| Decision Tree | max_depth | [None, 10, 30] |
|  | min_samples_split | [2, 5, 10] |
|  | min_samples_leaf | [1, 2, 4] |
|  | criterion | ['gini', 'entropy'] |
| Random Forest | n_estimators | [200, 500, 800] |
|  | max_depth | [None, 10, 30] |
|  | min_samples_split | [2, 5, 10] |
|  | min_samples_leaf | [1, 2] |
| Extra Trees | n_estimators | [200, 500, 800] |
|  | max_depth | [None, 10, 30] |
|  | min_samples_split | [2, 5, 10] |
|  | min_samples_leaf | [1, 2] |
|  | max_features | ['sqrt', 'log2', None] |
| XGBoost | n_estimators | [200, 500, 800] |
|  | max_depth | [3, 6, 9] |
|  | learning_rate | [0.01, 0.05, 0.1] |
|  | subsample | [0.8, 1.0] |
| Support Vector Machine | C (regularization parameter) | [0.1, 1, 10] |
|  | kernel | ['linear', 'rbf', 'poly'] |
|  | gamma | ['scale', 0.1, 0.01] |
|  | class_weight | [None, 'balanced'] |
| K-Nearest Neighbors | n_neighbors | [5, 15, 30] |
|  | weights | ['uniform', 'distance'] |
|  | p | [1, 2, 3] |
|  | leaf_size | [10, 30, 50] |
| Neural Network (MLP) | hidden_layer_sizes | [(32,), (64,), (128,)] |
|  | activation | ['relu', 'tanh', 'logistic'] |
|  | alpha (L2 penalty) | [1e-5, 1e-4, 1e-3] |
|  | learning_rate_init | [0.001, 0.01] |
| Gaussian Naive Bayes | var_smoothing | [1e-12, 1e-9, 1e-6] |

Note: All models were trained using a reproducible preprocessing-and-modeling pipeline. Categorical variables were one-hot encoded; continuous variables were standardized (z-score) and missing values were imputed using statistics computed from the training folds only, then applied to the corresponding validation/test data within the same pipeline to prevent data leakage. Hyperparameters were tuned via grid search with 10-fold cross-validation on the training set using a fixed random seed (random_state=33). To ensure comparability across models, we used a consistent low/medium/high value design (log-spaced for scale parameters) and a uniform maximum grid-search budget of 54 candidate configurations per model where applicable; Gaussian Naive Bayes has a single tunable parameter and was tuned using the same three-level log-spaced design.
